# The RIC COVID-19 Recruitment & Retention Toolkit: A community-informed resource of recruitment tools and strategies for clinical trial investigators

**DOI:** 10.1017/cts.2022.429

**Published:** 2022-07-19

**Authors:** Nan Kennedy, Julia Dunagan, Leslie R. Boone, Tiffany Israel, Stephanie A. Mayers, Leah Dunkel, Sarah Cook, Pamela Pimentel, Terri L. Edwards, Mary Stroud, Consuelo H. Wilkins, Paul A. Harris

**Affiliations:** 1 Vanderbilt Institute for Clinical and Translational Research, Vanderbilt University Medical Center, Nashville, TN, USA; 2 Community Research Consultant, Santa Ana, CA, USA; 3 Community Advisory Board, Recruitment Innovation Center, Nashville, TN, USA; 4 Department of Medicine, Vanderbilt University Medical Center, Nashville, TN, USA; 5 Department of Internal Medicine, Meharry Medical College, Nashville, TN, USA; 6 Office of Health Equity, Vanderbilt University Medical Center, Nashville, TN, USA; 7 Department of Biomedical Informatics, Vanderbilt University Medical Center, Nashville, TN, USA

**Keywords:** COVID-19, clinical trial recruitment, participant retention, toolkit, community engagement

## Abstract

The Recruitment Innovation Center (RIC) has created a toolkit of novel strategies to engage potential participants in response to recruitment and retention challenges associated with COVID-19 studies. The toolkit contains pragmatic, generalizable resources to help research teams increase awareness of clinical trials and opportunities to participate; produce culturally sensitive and engaging recruitment materials; improve consent and return of results processes; and enhance recruitment of individuals from populations disproportionately impacted by COVID-19. This resource, the “RIC COVID-19 Recruitment and Retention Toolkit,” is available free online. We describe the toolkit and the community feedback used to author and curate this resource.

## Introduction

Early data from the SARS-CoV-2 pandemic demonstrated a disproportionate impact of COVID-19 infection, hospitalization, and mortality rates among Black, Hispanic, and Asian communities compared to the white population [1]. Moreover, the pandemic altered the clinical trial landscape and created challenges in participant recruitment and retention for both COVID- and non-COVID-related studies. Innovative solutions designed to make trials safer and less burdensome to participants during the pandemic [2] can also improve clinical trial recruitment going forward, especially with respect to disadvantaged populations that already face greater barriers to clinical trial participation.

**Fig. 1.**
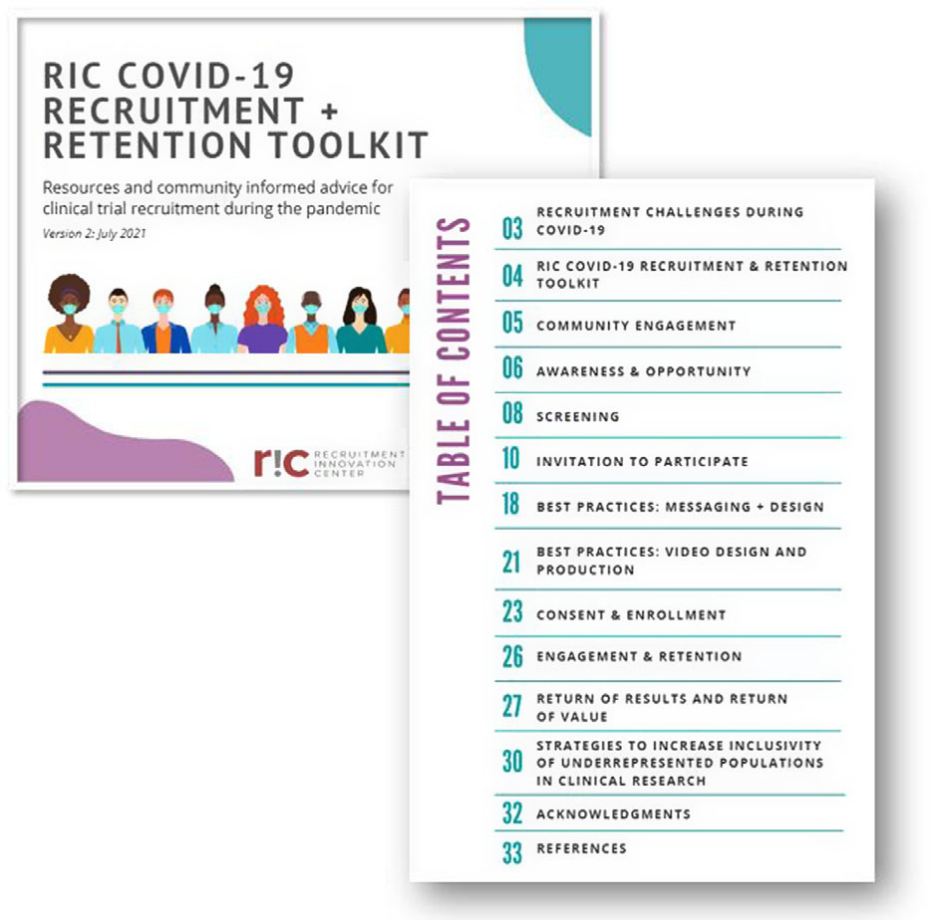
The RIC COVID-19 Recruitment & Retention Toolkit. Key: RIC – Recruitment Innovation Center.

The Vanderbilt Recruitment Innovation Center (RIC) [3], part of the Trial Innovation Network (TIN) [4,5], is our nation’s only federally funded center charged with developing and field-testing new strategies and methods to catalyze multisite clinical trial recruitment. The RIC’s transdisciplinary team of experts in community engagement, recruitment material development, and informatics methods has consulted on recruitment strategies for over 36 COVID-19 treatment trials. This extensive experience, gleaned in a short period of time, led to development of new strategies and tools that specifically address recruitment and retention challenges unique to, or exacerbated by, the pandemic. These methods, guidelines, and community-informed recommendations have been compiled into the “RIC COVID-19 Recruitment and Retention Toolkit” (Fig. 1). This toolkit, free and easily accessible on the TIN website [6], seeks to expand capacity and expertise for study teams as they strive to conduct trials in a manner that is safe, trustworthy, and respectful of all participants.

This paper describes how the toolkit was created and disseminated. It also provides guidance to others who may be developing research-related toolkits on how to use community feedback to ensure that guidelines and strategies they develop are culturally sensitive and relevant to racial and ethnic populations they seek to include as research participants.

## Methods

**Fig. 2.**
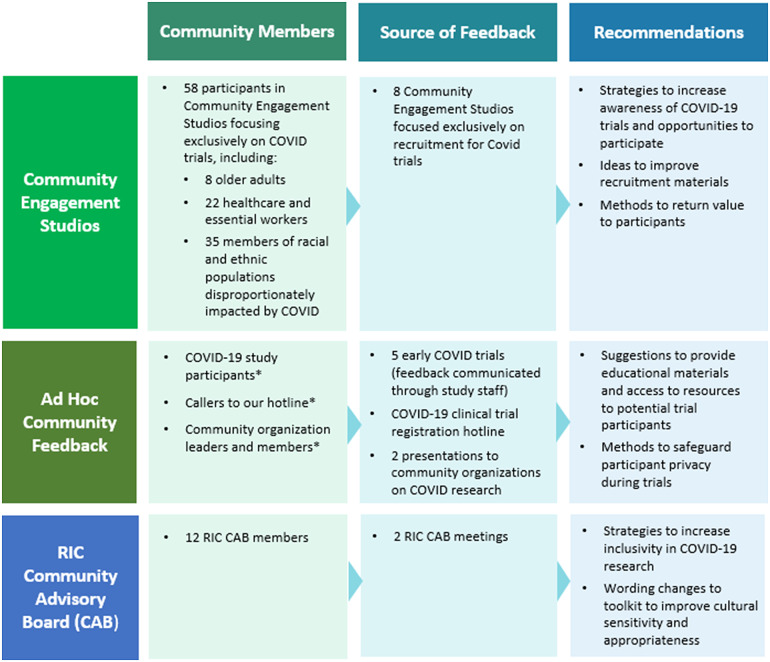
Community feedback used to inform the development of the RIC COVID-19 Recruitment and Retention Toolkit. Key: RIC – Recruitment Innovation Center; CAB – Community Advisory Board. *The number of community members who offered informal suggestions through these channels was not tallied.

The RIC consulted on design and implementation for 36 COVID-related clinical trials in 2020 and 2021. In the process, we developed and refined new tools and methods for recruitment and retention during COVID-19. We compiled these resources into a toolkit, which includes screening and consenting tools, templates for clinician-facing study information sheets and for returning study results to participants, and best practices guidelines for designing participant-facing websites, recruitment materials, and videos. To augment these resources and further inform their development and usefulness, the RIC also compiled community input on clinical trial recruitment and retention practices, by means of both formal and informal discussions with members and leaders of diverse communities. We gathered and organized the feedback, then synthesized and categorized the accumulated information. Figure 2 details the types and numbers of community members involved, the forms of interactions, and the areas in which their advice and suggestions informed the toolkit.

### Community Engagement

The Community Engagement (CE) Studio model is a structured approach to eliciting feedback from diverse groups of community stakeholders, who are considered experts in the topic under study by means of their lived experiences [8]. To leverage the extensive community engagement work the RIC had already completed, we identified 89 past CE Studios that engaged a total of 1357 diverse community members, for which the primary focus was recruitment and retention. Of these 89 CE Studios, 8 recruited individuals specifically from groups disproportionately impacted by COVID-19, including persons from underserved racial and ethnic groups, older adults, healthcare and essential workers, and individuals living in COVID-19 outbreak hotspots. These eight Studios focused on community members’ thoughts and opinions on implementation of planned COVID-19 research. To locate and garner the ideas of greatest relevancy to COVID-19 recruitment and retention, we read compilations of findings from each of the 89 Studios. Next, we aggregated the recommendations by key demographics and synthesized the information into toolkit categories (see Toolkit Description and Content).

### Ad Hoc Community Feedback

We also received informal, anecdotal feedback from other community sources, including participants in five early COVID-19 trials (commenting through study staff), ad hoc conversations with community members who called our COVID-19 clinical trial registration hotline, and at two presentations on current COVID-19 research to community organizations. This feedback, while not part of a formal process, uncovered real-world examples of barriers to COVID-19 trial participation that we deemed valuable to share with other researchers. For example, we learned the importance of establishing methods to safeguard participant privacy during COVID-19 trials; designing and providing easily understood educational materials about the virus and treatment; and being prepared to assist potential participants seeking medical care. This anecdotal feedback is labeled as “Lessons Learned” in the toolkit.

### Community Advisory Board (CAB)

Additionally, as we developed the toolkit, we gathered iterative input from the RIC Community Advisory Board (CAB) at two consultative meetings. The RIC CAB, a panel of 12 members representing diverse communities across the United States, regularly provides recommendations and guidance to the RIC team to ensure that our strategies and materials reflect the needs, priorities, and values of the wider community. Input from the RIC CAB led to wording changes that made the toolkit more culturally sensitive and participant-focused, and was used to develop a summary of 20 strategies to increase inclusivity of populations disproportionately impacted by COVID-19 and underrepresented in clinical research.

## Toolkit Description and Content

**Fig. 3.**
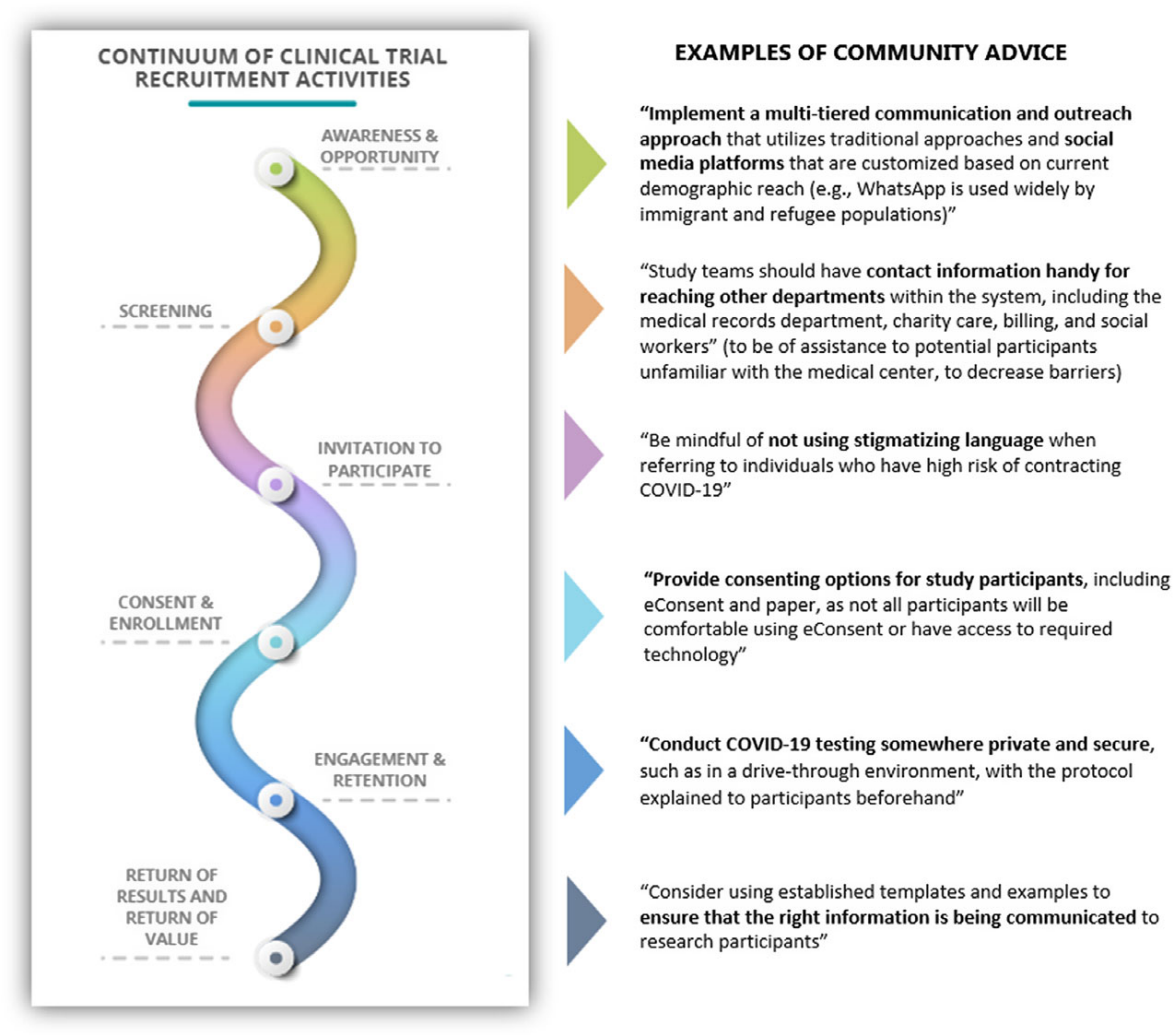
Continuum of clinical trial recruitment activities and examples of community feedback to support those activities.

The toolkit is organized by steps a researcher might take to optimize recruitment, retention, and return of value during the lifecycle of a participant’s journey – from study awareness and opportunity, the invitation to participate, and consenting, through continuing engagement and retention, and finishing with return of study results or study value to participants. Figure 3 shows this continuum and examples of recommendations received from the community on how best to implement each of these steps. The entire compendium of stakeholder advice can be found in the toolkit.

### Awareness and Opportunity

Community experts offered a range of ideas on expanding awareness of COVID-19 studies and increasing the opportunity to participate by reducing or eliminating barriers to participation. These included collaborating with local and state government and health agency leaders to promote studies in press announcements; partnering with local organizations and companies such as unions, restaurants, and childcare centers to expand outreach; and approaching patients upon admission for hospital-based studies to allow ample time for decision-making.

### Screening

The toolkit describes the development of a COVID-19 Research Data Mart [9] for efficient participant screening. The Data Mart is a REDCap [10] (Research Electronic Data Capture) database of COVID-19-positive patients who have agreed to direct contact about COVID-19 research opportunities. Since VUMC operates as an opt-in institution for research contact, a COVID-specific workflow was initiated. All patients who receive a COVID-19 test at a VUMC facility receive a "Consent to Contact" message via email (default) or SMS message. Custom logic reflecting study-specific inclusion and exclusion criteria facilitates real-time matching of active studies with potentially eligible patients. Approximately 3000 individuals who tested positive for COVID-19 responded favorably to being contacted directly and were matched to studies in the COVID-19 Data Mart. While the Data Mart is proprietary to Vanderbilt, we published a paper [9] to disseminate our efforts broadly. Other institutions can use REDCap (free to academic research institutions) and our published process to pattern their own efforts after ours.

### Invitation to Participate

Recruitment materials, including tailored content and design geared toward specific audiences, can have a major impact on recruitment success. Using best practices generated through community stakeholder feedback, the RIC developed or provided input on multiple, varied COVID-19 study materials such as flyers with QR Codes linked to study recruitment websites, study fact sheets and consent discussion guides, mobile apps for clinicians to share information with their patients about relevant studies, social media messaging, and educational and instructional videos. Examples of these materials are displayed in the toolkit.

An example of how the RIC incorporated community feedback to develop materials specific to recruitment during COVID-19 is found in the Passive Immunity Trial for Our Nation (Pass It On) Study. Pass It On was a multi-center clinical trial evaluating the use of convalescent plasma in hospitalized COVID-19 patients. Community feedback, especially from racially and ethnically diverse communities, was collected and applied to craft the trial’s public-facing study website, social media presence, and videos explaining the trial and its importance. Pass It On enrolled 974 patients across 26 trial sites, with 40% of study participants identifying as a member of a marginalized racial or ethnic population. Examples of the successful recruitment materials from this study are included in the toolkit.

### Consent and Enrollment

In challenging clinical trial circumstances, such as those confronting research teams during the COVID-19 pandemic, electronic consenting (eConsent [11]) is especially salient, as it can facilitate consenting accomplished remotely. The eConsent process has been enriched by recommendations from community experts and can be further enhanced with customization ideas from the toolkit. Feedback from diverse groups of community stakeholders informed formatting, tools, and recommendations relating to eConsent, including integration of photos and videos, additional tools to facilitate consenting in multiple languages, and options to improve accessibility (e.g., option for consent language to be read aloud).

### Engagement and Retention

Participant engagement and retention are necessary for achieving successful study completion goals. Expert advice from the community stresses consistent communication with participants, an emphasis on the value of the study, and the provision of fair compensation as critical methods to retain participants.

### Return of Results and Return of Value

Researchers have an ethical obligation to share study findings with participants, who are partners in the research. Participants have consistently expressed a desire to receive research results for studies they take part in [12,13], and they prefer that these findings be presented to them in an engaging format [10]. The toolkit contains an example of a study result graphic and a link to study results templates. In addition, other types of information that participants value just as highly as results [14–16] are documented in the toolkit.

### Toolkit Format and Dissemination

The toolkit was designed to be visually appealing, with color graphics, bulleted advice from community experts, and sidebars of “Lessons Learned.” Investigators can navigate easily through the document to locate needed information. Some of the RIC’s existing tools and methods were adapted to suit particular recruitment needs during the pandemic, while others were newly conceived and developed by the RIC to address specific challenges and gaps.

The COVID-19 Recruitment and Retention Toolkit was posted on February 5, 2021, on the TIN website in the TIN Toolbox. It has been widely disseminated to researchers through national webinars and announcements on the TIN website. An updated toolkit, containing additional materials and improvements in verbiage and formatting, was disseminated in July 2021. Since its initial posting, the toolkit has been viewed or downloaded 925 times (as of June 30, 2022).

## Discussion

Throughout the toolkit, particular attention is paid to modalities for supporting inclusion of all populations in COVID-19 clinical research, especially members of racial and ethnic populations most heavily impacted by COVID-19 and individuals with limited English-language proficiency. By including members of the non-academic community on research teams or involving community health workers and community partners as “bridge alternatives” or liaisons, trust in researchers can be built organically. The toolkit contains a compilation of recommendations geared specifically toward the goal of helping research teams view recruitment activities through the lens of diversity.

Toolkits consisting of guidelines, strategies, and templates have become popular vehicles for distributing information to healthcare providers and the public alike [17]. The development of toolkits designed to disseminate clinical trial methods and strategies to investigators has also begun to take hold [18,19], including those with the express purpose of increasing involvement of participants and community-based organizations in the research process [20,21]. To our knowledge, however, no published toolkit for clinical research has used community feedback as a core process for informing a toolkit’s materials. The RIC COVID-19 Recruitment & Retention Toolkit demonstrates that using community engagement principles and informal feedback processes to yield insights and suggest new strategies can be both valuable and attainable.

### Limitations and Evaluations

Because the toolkit was developed rapidly during the pandemic, no formal evaluation has been conducted to evaluate investigator success in increasing recruitment and retention through use of the toolkit’s materials. However, we currently collect institutional affiliation and contact information from individuals who download the toolkit; we plan to conduct a survey to evaluate toolkit usage and impact on enhancing recruitment and retention. Findings will be used to reveal gaps in knowledge and implementation and inform future iterations of the toolkit, as we expect to update and expand it over time as more community feedback becomes available.

## Conclusion

The challenging clinical trial milieu necessitated by COVID-related concerns and restrictions has forced clinical trial investigators to devise new strategies to conduct research in a way that feels safe, convenient, and attractive to potential participants from diverse backgrounds and circumstances. The RIC COVID-19 Recruitment & Retention Toolkit was created to provide researchers with novel strategies and resources to help them achieve their recruitment and retention goals, while also motivating them to turn to community feedback, especially from populations disproportionately affected by a condition or disease, more readily and enthusiastically when developing their own research plans, methods, and toolkits.
